# *The Organon* and Materia Medica Club of the Bay Cities of California*In consequence of the failure of this report to arrive on time, it was not possible to publish it in its proper order with the other reports.

**Published:** 1896-02

**Authors:** W. E. Ledyard

**Affiliations:** Secretary


### *THE ORGANON* AND MATERIA MEDICA CLUB OF THE BAY CITIES OF CALIFORNIA.*

The regular semi-monthly meeting was held at the office of Dr. A. McNeil, 784 Van Ness Avenue, San Francisco, June 21st, 1895.

Members present were: Drs. A. McNeil, G. J. Auger, George H. Martin, S. E. Chapman, J. M. Selfridge, M. T. Wilson, and W. E. Ledyard.

The meeting was called to order at 8 P. M. by the President, Dr. J. M. Selfridge.

The Secretary, Dr. Ledyard, read the minutes of the meetings held April 19th, May 17th, and June 7th, 1895, all of which, after some slight corrections, were approved.

Some little discussion took place in regard to the publication of the minutes of the meetings, Dr. Selfridge stating that the *Medical Advance* and THE HOMŒOPATHIC PHYSICIAN wanted to publish them.

Dr. McNeil said that he heard from the *Medical Advance* that unless they could have the exclusive right to the minutes of every meeting they were not particularly anxious to publish them at all, and hence he made a motion that the minutes be sent to THE HOMŒOPATHIC PHYSICIAN, commencing with the minutes of the meeting of June 7th. This was seconded by Dr. Ledyard. Carried.

The President then said that he thought a vote of thanks was due Dr. Eleanor F. Martin for the splendid report she had made of the previous meeting, as it was by far the best report the Society had ever had, whereupon Dr. Ledyard moved that a vote of thanks be tendered Dr. Eleanor F. Martin for the work she had done, which was seconded by Drs. Wilson and McNeil, and carried.

Dr. Selfridge then asked what decision the members had

---

* In consequence of the failure of this report to arrive on time, it was not possible to publish it in its proper order with the other reports.

arrived at in regard to the construction they placed upon Section 80 of *The Organon* at the last meeting.

Dr. NcNeil said that Hahnemann explained it in Section 204.

The President then appointed Dr. Martin reader of the evening, who commenced at Section 201 and continued to Section 205.

DISCUSSIONS.

Dr. Chapman—How do you harmonize Section 204 with Section 80?

Dr. Martin—If we read Section 80 carefully this will be explained to us. He then read Section 80. The word "other" in this section explains the whole meaning as intended by Hahnemann. That there are syphilitic and sycotic miasms, and that all other diseases, with the exception of these two mentioned, have for their fundamental cause *psora.*

Dr. McNeil—Do I understand Dr. Martin to say that he gives up the ground which he maintained at the last meeting as regards psora being the fundamental cause of *all* diseases, syphilis and sycosis included?

Dr. Martin—Yes. Psora is the *greatest* of these miasms. According to Section 80, the word "other" changes the construction which I placed upon the sentence at the last meeting. This section admits the existence of sycosis and syphilis, but says that psora is the basis of all *other* diseases.

Dr. McNeil—In reading this there is one point that has always struck me—that Hahnemann did not bring out forcibly enough the hereditary theory of psora at the time he was writing. Even to-day, in the polyclinics of Europe, you will find that one-third of all dermatological cases are itch. Hahnemann said nothing of the *acarus scabiei.*

Dr. Chapman—Hahnemann wrote the psoric theory before the *acarus scabiei* was discovered.

Dr. McNeil—If you read the article by Carroll Dunham on psora, you will find that he makes the statement that Hahnemann must have been aware of the *acarus scabiei,* and brings out more direct statements regarding it. It was discovered long before he wrote the psoric theory.

Dr. Auger—I would like to ask Dr. McNeil if psora is the primary though invisible cause of all skin diseases? Are there no exceptions?

Dr. McNeil—No, except syphilis and sycosis. You have reference to parasitic diseases as exceptions. The parasites cannot live unless psora is present. It has been proven that a person may have the itch without the *acarus scabiei* being present. Itch may be communicated. Take out an acarus, put it under the skin, then hunt for it, and you will not be able to find it, and yet you will have a full case of itch. Every one is not susceptible to *acarus scabiei.*

Dr. Chapman—Do I understand Dr. McNeil to say that every case of itch is absolutely from a psoric taint?

Dr. McNeil—The primary manifestation of psora is the itch. An illustration: Suppose the bacillus syphili is communicated to a person (they are usually in the amorphus condition), there will be a number of skin diseases developed, different, and yet they will all be classed under syphilis. All these manifestations are of one disease. The primary manifestation of psora is itch. There are twenty or fifty skin diseases developed from the bacillus syphili, as gummata, ecthyma, erythema, chancre, etc., and yet they are all classified under the name of syphilis.

Dr. Ledyard—I have a family under my care whom I have been treating for some time. Another family moved into the same house with them, who had *pediculus capitis.* The two families were frequently together, and yet neither the parents or children of the family I had been treating took it.

Dr. McNeil—I have a case of a German woman, fine looking, clear-skinned, plump, and rosy. She had itch when a child, which was cured (?), and after some years *epilepsy developed.* She was then sent to the country to work as a peasant, with the result that the attacks of epilepsy stopped, but were followed by terrible headaches. She came to me, and I saw that her symptoms were clearly Sulphur. She had burning on the top of the head, soles of the feet, and palms of the hands, with also *a few*

*vesicles on the hands.* She married soon after, and has now a boy six years of age. Her husband, who was consumptive, died soon after the birth of the boy. I cured all of her symptoms with Sulphur. I think that the itch was the cause of all these other conditions.

Dr. Ledyard—Did the itch come to the surface again?

Dr. McNeil—No; it did not appear again, except the few vesicles on the hands.

Upon motion of Dr. Wilson, seconded by Dr. McNeil, the meeting then adjourned to meet the first Friday in July, at the office of Dr. Ledyard, 223 Post Street, San Francisco, when the reading of *The Organon* would be commenced at Section 205.

W. E. LEDYARD, *Secretary.*

Reported by Eleanor F. Martin, M. D.

---

